# Molecular Survey for Major Canine Enteric Viral Pathogens in Wild Carnivores, Northwestern Italy

**DOI:** 10.3390/vetsci12090814

**Published:** 2025-08-26

**Authors:** Vittorio Sarchese, Federica Di Profio, Serena Robetto, Riccardo Orusa, Beatrice Vuillermoz, Francesco Pellegrini, Fulvio Marsilio, Vito Martella, Barbara Di Martino

**Affiliations:** 1 Department of Veterinary Medicine, Università degli Studi di Teramo, Località Piano D’Accio, 64100 Teramo, Italy; fdiprofio@unite.it (F.D.P.); fmarsilio@unite.it (F.M.); bdimartino@unite.it (B.D.M.); 2 Centro di Referenza Nazionale per le Malattie degli Animali Selvatici (CeRMAS), Istituto Zooprofilattico Sperimentale del Piemonte, della Liguria e della Valle d’Aosta, 11020 Quart, Italy; serena.robetto@izsplv.it (S.R.); riccardo.orusa@izsplv.it (R.O.); beatrice.vuillermoz@izsplv.it (B.V.); 3 Department of Veterinary Medicine, Università Aldo Moro di Bari, S.p. per Casamassima Km3, Valenzano, 70010 Bari, Italy; francesco.pellegrini@uniba.it (F.P.); vito.martella@uniba.it (V.M.); 4 Department of Pharmacology and Toxicology, University of Veterinary Medicine, 1078 Budapest, Hungary

**Keywords:** wild carnivores, viruses, nanopore sequencing, viral surveillance

## Abstract

Wild carnivores can harbor pathogens affecting wildlife conservation and domestic animal health. This study surveyed major viral pathogens in free-ranging wolves, red foxes, stone martens, and Eurasian badgers in Northwestern Italy. Duodenal samples from 140 carcasses were screened by consensus PCR for members of the species *Protoparvovirus carnivoran1* and for canine adenoviruses (CAdV-1/2). PCR-positive samples underwent sequence-independent amplification and Oxford Nanopore sequencing. Canine parvovirus type 2 (CPV-2) and feline panleukopenia virus (FPV) DNAs were identified in three wolves (6.4%) and one badger (4.3%), whereas CAdV-1 was detected in one red fox (1.8%). Nanopore sequencing yielded near-complete genomes of two CPV-new 2a, one CPV-2c, and one FPV strains, along with partial CAdV-1 sequences. Furthermore, the complete genome of a canine circovirus (CaCV) strain in co-infection with a CPV-2c-positive wolf and partial sequences of a canine kobuvirus (CaKoV) strain were also obtained. Phylogenetic analysis placed these viruses within known European lineages and linked them to domestic and wild hosts. These findings revealed the circulation of multiple viral pathogens among wild carnivores, reflecting ongoing cross-species spillover. Continuing molecular surveillance at the wildlife–domestic interface is recommended.

## Simple Summary

Wildlife animals could act as a source of viral pathogens for pets, complicating the ecology of these viruses and the plans for controlling the disease. In particular, canine protoparvovirus and canine adenovirus are considered major pathogens of dogs, and the vaccines for the prevention of canine protoparvovirus and adenovirus-associated diseases are considered core vaccines. This study examined the occurrence of these viral pathogens in wild carnivores in Northwestern Italy. The investigation included free-ranging wolves, red foxes, stone martens, and Eurasian badgers, combining consensus PCR assays and third-generation sequencing. Carnivore protoparvoviruses were identified in three wolves and one badger, while canine adenovirus type 1 was detected in one fox. The results confirm the circulation of major viral pathogens among wild canids and mustelids. The study underscores the risk of cross-species transmission at the domestic–wildlife interface and highlights the value of molecular surveillance for emerging pathogens in wildlife populations.

## 1. Introduction

The order *Carnivora* comprises over 290 species and plays a crucial role in maintaining ecological balance by regulating populations of herbivores and smaller carnivores, facilitating seed dispersal, and preserving local vegetation dynamics [1,2]. However, these ecological contributions are increasingly threatened by several factors, including habitat degradation, environmental changes, and infectious diseases [3]. In particular, infectious diseases pose a significant threat to wild carnivores in increasingly anthropized landscapes, resulting in the growth in domestic animal populations around protected areas and greater opportunities for inter-species disease transmission [4]. Indeed, carnivores at the human–wildlife interface are known to influence the epidemiology of domestic carnivore viruses [5]. Specifically, wild carnivores face a heightened risk of viral transmission due to their close phylogenetic relationship with domestic dogs and cats [6]. Increased contact between these groups fosters pathogen exchange among related hosts, such as species within the *Canidae* and *Felidae* families [7,8].

Among these pathogens, members of the *Protoparvovirus carnivoran1* species (family *Parvoviridae*, genus *Protoparvovirus*), which include canine parvovirus type 2 (CPV-2) and feline panleukopenia virus (FPV) [9], are of particular concern. CPV-2 variants (CPV-2a, CPV-2b, CPV-2c) and FPV are often associated with cross-species transmission from domestic to wild carnivores, with molecular studies confirming their widespread circulation [10,11,12]. In Europe, CPV-2 has been identified in different wild animals, like foxes (*Vulpes vulpes*) [13,14,15], wolves (*Canis lupus*) [13,14,15,16,17], stone martens (*Martes foina*) [13,14,18], Eurasian badgers (*Meles meles*) [14,15,19], and Eurasian otters (*Lutra lutra*) [20], in a common genet (*Genetta genetta*), and in a European wildcat (*Felis silvestris*) [13]. On the other hand, FPV has been detected in foxes [14,15,18,21], Eurasian badgers [13,14,15,22], stone martens [13,15], golden jackals (*Canis aureus*) [22], Eurasian lynx (*Lynx lynx*) [23], Iberian lynx (*Lynx pardinus*) [24], wolves, crested porcupines (*Hystrix cristata*), and Marsican brown bears (*Ursus arctos marsicanus*) [15].

In addition to CPV-2 and FPV, canine adenoviruses (CAdVs) are important pathogens of domestic dogs and are able to infect different species of wild carnivores, although their ecology and impact remain poorly investigated. CAdVs are classified in the genus *Mastadenovirus* within the family *Adenoviridae* [25]. Two distinct types of the virus are known, canine adenovirus type 1 (CAdV-1) and canine adenovirus type 2 (CAdV-2), causing infectious canine hepatitis (ICH) and kennel cough in dogs, respectively [26]. CAdV-1 was described in silver foxes mainly associated with encephalitis [27] and has been since then identified in diseased and apparently healthy wild carnivores [28,29,30,31,32,33,34]. Likewise, CAdV-2 has been documented in foxes [21] and wolves in Italy [35] and in wild raccoon dogs (*Nyctereutes procyonoides*) in Korea [36], but its epidemiological role and pathogenicity are not clear yet.

In this study, by employing a combination of molecular diagnostic approaches and third-generation sequencing with the Oxford Nanopore Technologies (ONT) platform, we investigated the presence of these major canine viral pathogens in free-ranging carnivore populations from Northwestern Italy.

## 2. Materials and Methods

### 2.1. Sampling

Molecular investigations were performed on archived duodenal samples collected from a total of 140 wild carnivores found dead, including 47 wolves (*Canis lupus*), 55 foxes (*Vulpes vulpes*), 15 stone martens (*Martes foina*), and 23 Eurasian badgers (*Meles meles*), between February 2021 and April 2022. Sampling was conducted in Northwestern Italy, specifically in the Piemonte and Valle d’Aosta regions, as part of a national passive surveillance program carried out by the National Reference Centre for Wild Animal Diseases (CeRMAS-IZS PLV).

### 2.2. Nucleic Acids Extraction and Molecular Investigations

Duodenal tissues (1g) were homogenized using a Mini Bead Mill (VWR International Srl, Milan, Italy) in 7 mL of hard-tissue homogenization tubes containing 4 mL of phosphate-buffered saline (PBS, 0.15 M, pH 7.2). To enhance tissue breakdown and promote virion release into the solution, 0.5 mL of proteinase K (PK) at a concentration of 100 μg/mL (1 mg/mL) was added. The samples were incubated at 37 °C for 3 h with agitation at 320 rpm. To inactivate the PK, the reaction mixture was further incubated at 60 °C for 15 min. After centrifugation at 2500 rpm for 15 min, the supernatant was collected and stored at −20 °C. RNA and DNA were extracted from 0.5 mL aliquots of the duodenal and liver samples using TRIzol LS reagent (Invitrogen, Ltd., Paisley, UK), according to the manufacturer’s protocol. Nucleic acid extracts were successively processed with the OneStep PCR Inhibitor Removal Kit (Zymo Research, Irvine, CA, USA) to remove potential PCR inhibitors. All samples were screened for the presence of members of the species *Protoparvovirus carnivoran1* [37] and for CAdV-1/CAdV-2 [38].

RT-PCR and PCR assays were carried out using SuperScript™ One-Step RT-PCR (Invitrogen, Ltd., Paisley, UK) and GoTaq^®^ Green Master Mix 1 (Promega Italia S.r.l., Milan, Italy), respectively. Amplified products were separated by electrophoresis on 1.5% agarose gels and visualized under UV illumination following ethidium bromide staining. Bands of the expected size were excised from the gels, purified using a QIAquick gel extraction kit (Qiagen GmbH, Hilden, Germany), and sequenced bidirectionally using BigDye Terminator Cycle chemistry on an Applied Biosystems 3730 DNA Analyzer (Foster, CA, USA). Resulting sequences were compared against databases using BLAST (http://www.ncbi.nlm.nih.gov) and FASTA (http://www.ebi.ac.uk/fasta33) with default parameters to identify homologous sequences.

### 2.3. SISPA, ONT Library Preparation and Sequencing

A sequence-independent single-primer amplification (SISPA) method [39], combined with Oxford Nanopore Technologies (ONT) sequencing, was employed to characterize the virome of the PCR-positive duodenal samples. Additionally, for each animal species, total RNA and DNA were combined into pools consisting of five samples mixed in equal volumes. These pooled RNA and DNA samples were then subjected to SISPA amplification and ONT sequencing.

Briefly, 20 µL of extracted DNA was combined with 2.5 µL of NEBuffer™ 2 (New England Biolabs, Hitchin, UK), 1 µL of 10 mM dNTPs (Invitrogen, Ltd., Paisley, UK), and 2 µL of 10 µM primer FR26RV-N [40]. The mixture was initially incubated at 94 °C for 2 min, followed by cooling on ice for an additional 2 min. Subsequently, 0.5 µL (2.5 units) of Klenow Fragment (3′→5′ exo-) (New England Biolabs, Hitchin, UK) was added, and the reaction was incubated at 37 °C for 1 h. This process was repeated, and then an enzyme inactivation step was performed at 75 °C for 10 min.

For reverse transcription, DNase-treated RNA was converted into single-stranded cDNA using SuperScript IV Reverse Transcriptase (Invitrogen Ltd., Milan, Italy) with primers FR26RV-N [40] and FR40RV-T [41]. The synthesis of the second cDNA strand was completed using Klenow Fragment (3′→5′ exo-) (New England Biolabs, Hitchin, UK). A 5 µL aliquot of the resulting reaction was used as the template in a subsequent PCR amplification. The 50 µL PCR mixture included 10 µL of 5X Q5 Reaction Buffer (New England Biolabs, Hitchin, UK), 1 µL of dNTPs, 1 µL of 10 µM primer FR20RV [40], and 1 µL of Q5 High-Fidelity DNA Polymerase (0.04 U/µL; New England Biolabs, Hitchin, UK). The thermal cycling protocol consisted of an initial activation at 94 °C for 2 min, followed by 40 cycles of amplification (94 °C for 30 s, 48 °C for 30 s, 68 °C for 8 min), and a final extension at 68 °C for 8 min.

PCR products were purified using AMPure XP beads (Beckman Coulter, Indianapolis, IN, USA) and quantified with a Qubit 4.0 fluorometer using the dsDNA HS Assay Kit (Thermo Fisher Scientific, Waltham, MA, USA). Library preparation was carried out according to the manufacturer’s protocol, utilizing the PCR Barcoding Expansion 1–12 (EXP-PBC001) Kit and the Ligation Sequencing Kit (SQK-LSK-114) to enable multiplexing of samples on an R10 flow cell (FLO-MIN110D, ONT). Sequencing was performed on a MinION Mk1C device (ONT, Oxford, UK) for six hours. Real-time basecalling and demultiplexing during sequencing were carried out using the ONT GUPPY (v3.2.8) basecaller within MinKNOW software (v3.1.5). FastQ MinION files were processed with the online tool Genome Detective (https://www.genomedetective.com/db/ui/login) [42] for de novo assembly with default settings. Additionally, reference assembly was performed using the Minimap2 plugin in Geneious Prime software v.2021.2.2 (Biomatters Ltd., Auckland, New Zealand, https://www.geneious.com). Phylogenetic analysis was conducted by using Maximum Likelihood or neighbor-joining methods, with statistical support with bootstrapping of 1000 replicates in MEGA 11 software [43].

## 3. Results

### 3.1. Molecular Screening and Sanger Sequencing

The molecular screenings revealed positivity in five of the 140 animals tested, with an overall prevalence of 3.6% (5/140) (Table 1). In detail, *Protoparvovirus carnivoran1* DNA was identified in three intestinal samples of wolves (6.4%, 3/47) from the Piemonte region and in one enteric specimen of a Eurasian badger, 4.3% (1/23), collected in Valle d’Aosta. Additionally, CAdV-1 DNA was detected in one intestinal sample collected from a fox found dead in the Valle d’Aosta region (1.8%, 1/55), while all other wild carnivores tested negative. All animals tested negative for CAdV-2, and all stone marten samples tested negative.

All amplicons (Supplementary Figure S1) acquired through consensus PCR protocols were subjected to direct sequencing. For the *Protoparvovirus Carnivoran1* strategy [37], sequence analysis revealed that all sequences detected shared 97.7–100.0% nucleotide (nt) identity to each other. Two wolf samples (LI6/2021/ITA and LI8/2021/ITA) exhibited the highest nt identity (99.65–100%) with CPV-2a strains detected in Canada in wild raccoons (*Procyon lotor*) [44] and in a wild American mink (*Neovison vison*) [45], respectively, as well as with strains identified in dogs in Italy [22,46,47], Hungary [48], New Zealand [49], and in cats in China [50]. The sample LI9/2022/ITA exhibited the closest relationship (99.65–100% nt identity) with CPV-2c strains identified in dogs in Italy [22,51], in India [52], in China [53], in Vietnam [54], and in Zambia [55]. The badger positive sample (TI6/2022/ITA) showed the closest match (99.47–99.65% nt) to FPV strains identified in wild animals, including strains identified in China in a giant panda (*Ailuropoda melanoleuca*) [50,56], in a red panda (*Ailurus fulgens*) [50], in a captive tiger [57], and in a captive jaguar (unpublished), in the brain of a Iberian lynx in Spain [24], in strains isolated in Italy from a crested porcupine [15] and a golden jackal [22], and in domestic cats in Italy [46], and Spain [24].

Sequence analysis of the CAdV-1 DNA amplicon (GenBank accession no. PV955937) showed the highest sequence identity (99.5–100.0%) to CAdV-1 strains previously identified in wolves in Italy [30] and in France [31], in foxes in Italy [29] and in the United Kingdom [58], in European brown bears in Spain (*Ursus arctos arctos*) [32], in a badger in Italy [59], in a civet in India (*Civettictis civetta*) (unpublished), and domestic dogs from various regions, including Australia [60] and Italy [61].

### 3.2. ONT Sequencing

After enrichment using a SISPA protocol, the five positive samples (Wolf/LI6/2021/ITA, Wolf/LI8/2021/ITA, Wolf/LI9/2022/ITA, Badger/TI6/2022/ITA, Fox/VI9/2021/ITA) and pooled samples were processed for high-throughput sequencing with ONT. A total of 2,816,158 raw nanopore reads were generated, and low-quality sequencing data were filtered using a quality score threshold of eight. A total of 1,807,115 (64.17%) viral reads, with an average length of 1315 nt (range 110–5588 nt), form a total of 16 contigs (>100 nt) were assembled using the NCBI reference sequence and compared with viral sequences currently available on the GenBank database (Table 2).

We generated the complete coding regions of the genomes for the parvoviral DNA-positive samples. Specifically, strains CPV-2/Wolf/LI6/2021/ITA (PV955931), CPV-2/Wolf/LI8/2021/ITA (PV955932), and CPV-2/Wolf/LI9/2022/ITA (PV955933) each formed a single contig aligned to reference sequence NC001539, with 131,239, 489,633, and 831,287 reads assembled, respectively. Similarly, the FPV strain FPV/Badger/TI6/2022/ITA (PV955934) formed a single contig with 344,382 reads assembled from 399,962 total reads. In detail, for the CPV-2/Wolf/LI6/2021/ITA, CPV-2/Wolf/LI8/2021/ITA, and CPV-2/Wolf/LI9/2022/ITA strains, a genome sequence of 4492 nt was generated, including a partial 5′ untranslated region (UTR) (128 nt), the complete NS1 sequence (648 aa), the complete NS2 sequence (165 aa), the complete VP1 sequence (727 aa), the complete VP2 sequence (584 aa), and a partial 3′ UTR (95 nt). The genome coding sequence of the FPV/Badger/TI6/2022/ITA (4591 nt) strain, excluding the terminal UTR regions, was 4258 nt and exhibited two major ORFs. The left ORF, coding for NS1 and NS2, was 2007 nt, and the right ORF, encoding for VP1 and VP2, was 2185 nt. Deduced VP2 amino acid sequences were compared with CPV-2 and FPV reference strains, along with strains identified in wolves and badgers. Strains CPV-2/Wolf/LI6/2021/ITA and CPV-2/Wolf/LI8/2021/ITA exhibited identical VP2 sequences, aligning closely with the CPV-new 2a strain and characterized by the mutation 297-Ser to Ala. Additionally, strain CPV-2/Wolf/LI9/2022/ITA, closely related to strains classified as CPV-2c, was distinguished by the substitution of 426-Asn to Glu, a hallmark of CPV-2c strains. Sequence alignment highlighted two amino acid mutations in the badger capsid sequence compared to the reference FPV strain, 101-Ile to Thr and 103-Val to Ala.

**Figure 1:**
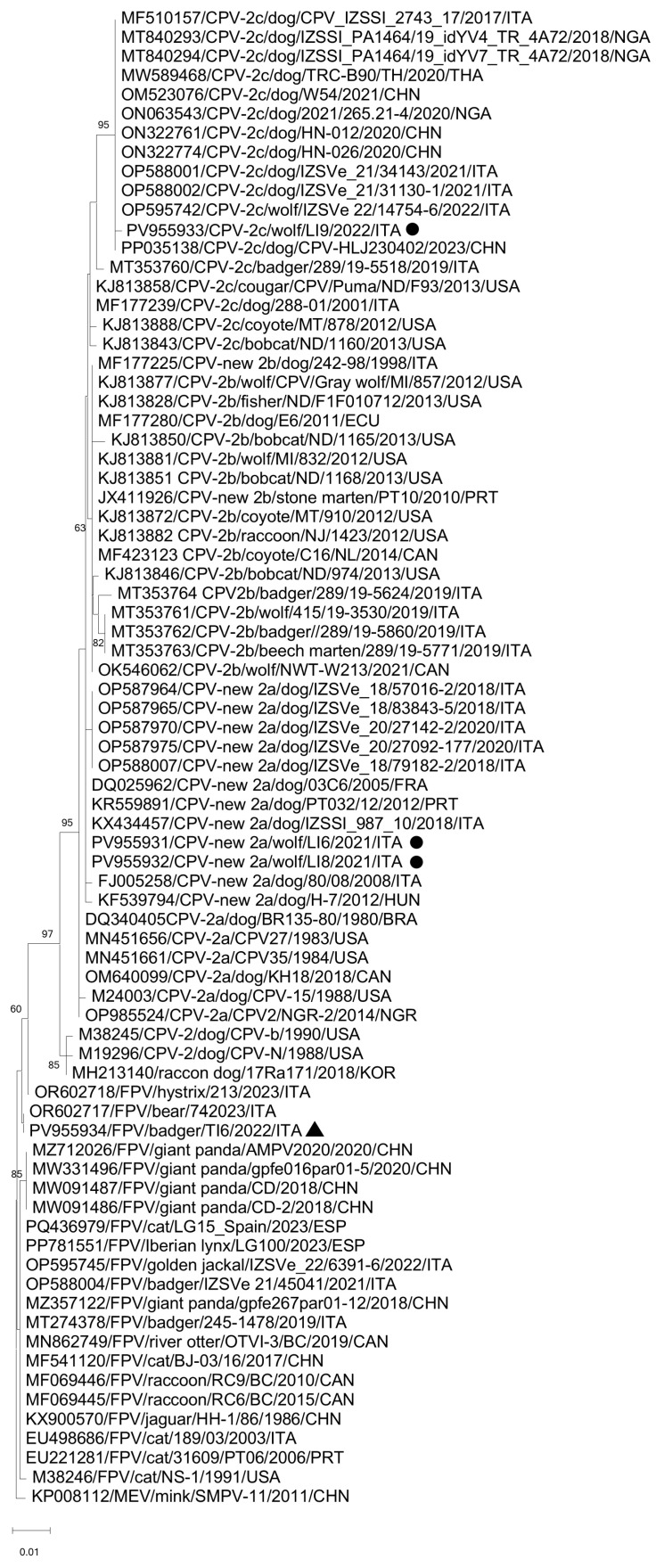
Phylogenetic tree based on the complete aa sequence of the VP2 gene of the CPV-2 and FPV strains detected in this study. A selection of cognate sequences of the species *Protoparvovirus carnivoran1* was retrieved from GenBank. Mink enteritis virus (SMPV-11) (GenBank accession n. KP008112) was used as an outgroup. The tree was generated using the neighbour-joining method and Jones-Taylor-Thornton substitution model, supplying statistical support with bootstrapping of 1000 replicates. Bootstrap values > 60% are shown. Black triangles and black circles indicate the FPV and CPV-2 strains, respectively, identified in this survey.

Phylogenetic analysis of the complete amino acid sequence of the VP2 gene indicated that the CPV-2 sequences from wolves were closely related to CPV-2 field isolates from Europe and America, sourced from various animal species. The FPV strain FPV/Badger/TI6/2022/ITA was segregated with the strain ITA/2023/bear/74, found in a Marsican brown bear in Central Italy [15], but separate from other FPV strains detected from different animal species (Figure 1).

In the sample LI9/2022/ITA, the combined SISPA/ONT approach generated an additional 588 reads forming a single contig of 2063 nt. This contig was assembled against the NC020904 strain, the prototype strain of the species *Canine circovirus*, genus *Circovirus*, family *Circoviridae* [62]. The genome corresponds to the complete genome of the strain CaCV/Wolf/LI9/2022/ITA (PV955935) and includes two main ORFs situated on complementary strands in opposite orientations. ORF1 (912 nt) is present on the virion strand, while ORF2 (813 nt) is located on the complementary strand of the replicative form. These ORFs encode the Rep (303 aa) and Cap (270 aa) proteins, respectively. Like other animal circoviruses, the genome of the Italian wolf strain features two intergenic non-coding regions, measuring 135 and 203 nt, positioned between the start and stop codons of the replicase and capsid protein genes. The 5′-intergenic region forms a thermodynamically stable stem-loop structure containing the conserved nucleotide sequence TAGTATTAC [63,64]. The nucleotide alignment between the complete genomic sequences of CaCV/Wolf/LI9/2022/ITA and the CaCV strains retrieved from the GenBank database displayed an overall nt identity ranging from 82.88% to 95.98%.

**Figure 2:**
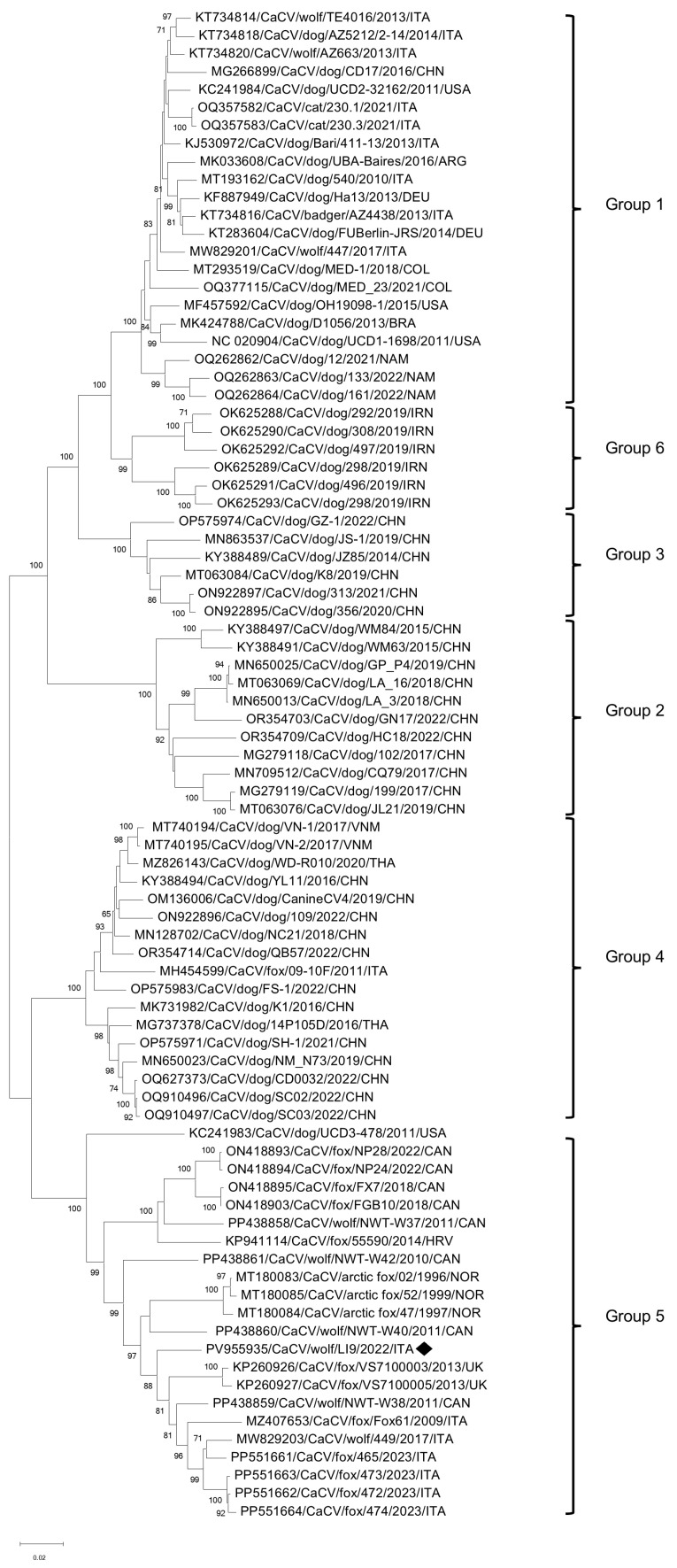
Unrooted phylogenetic tree was constructed based on the complete genome nucleotide sequences of CaCV strain obtained in this study and cognate strains retrieved from the GenBank database. The phylogeny was inferred using the Maximum Likelihood method under the General Time Reversible (GTR) model with gamma distribution and invariable sites. To assess the robustness of the tree, bootstrap analysis was performed with 1000 replicates, and bootstrap values ≥ 70% are indicated on the tree. The scale bar represents the estimated number of nucleotide substitutions. Black rhombus indicates the CaCV strain identified in this study. To the right of the figure, the groups evidenced in this study are indicated and correspond to proposed clusters [65,66].

Based on the complete genome nucleotide sequences, the unrooted phylogenetic tree (Figure 2) reveals the clustering of CaCV strains into six well-defined groups. Group 1 comprises strains identified in dogs, wolves, and a badger across Europe, America, and Asia. Groups 2, 3, and 4 include CaCVs detected in dogs from Asia and a red fox in Italy. Group 6 consists of sequences detected in Iranian dogs, while Group 5 encompasses CaCVs identified in wolves, and arctic and red foxes from various countries [65,66]. The sequence from this study clusters within Group 5 alongside strains isolated from foxes and wolves in Europe and in Canada.

For the sample Fox/VI9/2022/ITA, positive for CAdV-1 DNA, SISPA, and ONT, sequencing produced eight contigs ranging in length from 102 to 7124 nt. These contigs partially covered the E1A protein gene, the E1B-55K gene, and the Iva2 coding sequence, as well as the pTP gene, pV gene, and the genes encoding hexon, protease, DNA-binding protein (DBP), and 100k protein. Furthermore, contigs fully encompassed the complete coding sequences of the E1B-19K, the polymerase (pol) gene, and the genes encoding 33K, 22K, pVIII, E3, U exon, fiber, and E4 proteins. Sequence analyses revealed nt identities ranging from 98.9% to 100.0% when compared with previously detected CAdV-1 sequences from liver, blood, lung, and fecal samples of dogs and various wild carnivores and classified within the species *Mastadenovirus canidae* of the genus *Mastadenovirus* (family *Adenoviridae*) [25].

**Figure 3:**
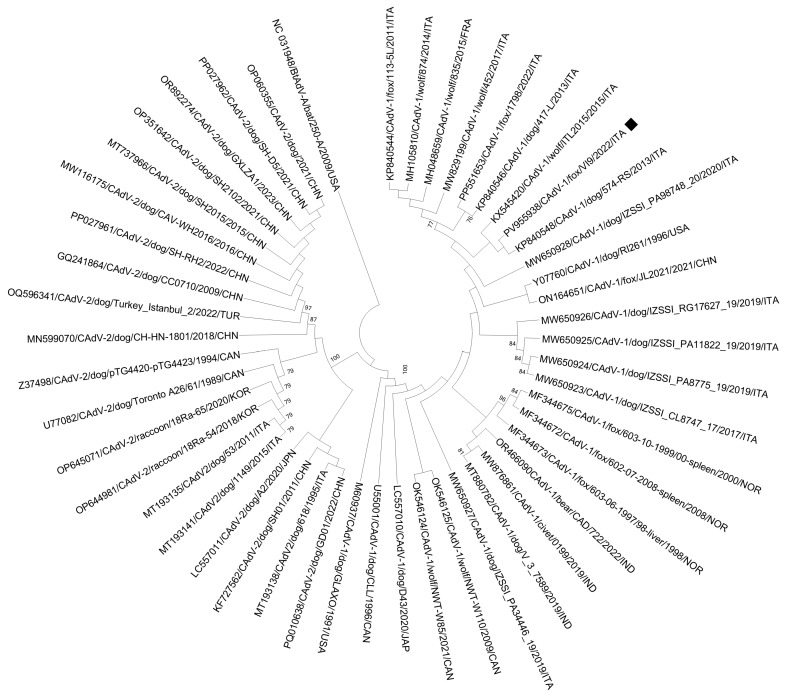
Phylogenetic analysis of CAdV-1/VI9/ITA/2022 strain. The tree was generated using the neighbor-joining method and the p-distance model, supplying statistical support with bootstrapping of 1000 replicates, and bootstrap values ≥ 70% are indicated on the tree. Black square indicates the CAdV-1 strain identified in this study. BtAdV (accession no. NC031948) was used as an outgroup.

In the phylogenetic tree constructed from the complete nucleotide sequences of the fiber gene (PV955938), strain CAdV-1/VI9/ITA/2022 strictly clustered with other CAdV-1 strains identified in Italy, in a dog rectal swab [67], and in a wolf liver sample [30] (Figure 3).

**Figure 4:**
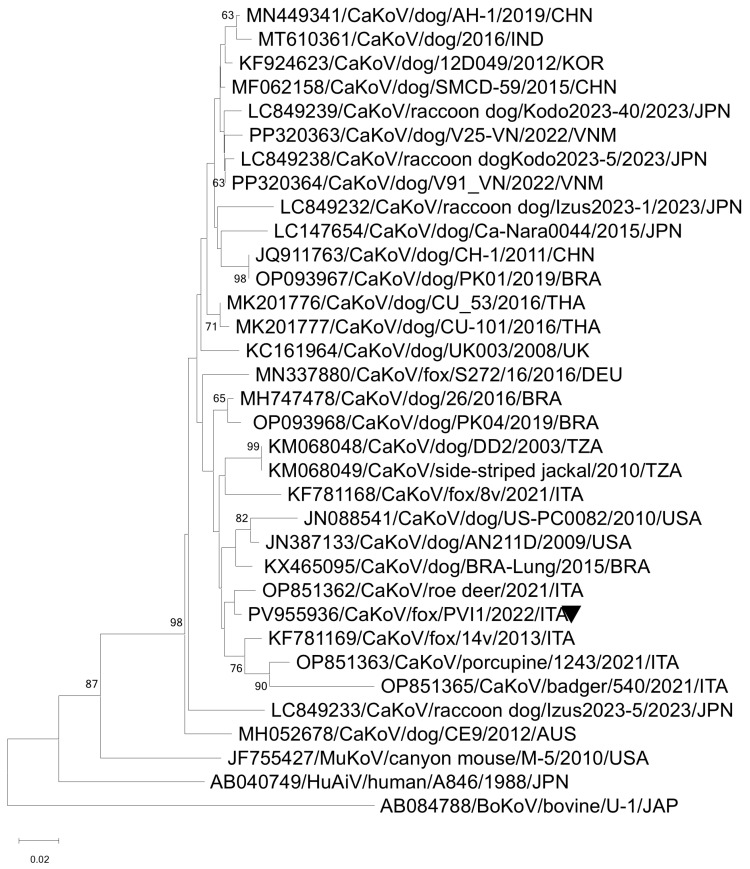
Neighbor-joining phylogenetic tree based on the nt sequence of the partial RdRp gene of the CaKoV strain detected in this study. The tree was generated using the neighbor-joining method and p-distance correction, with statistical support provided by bootstrapping with 1000 replicates. The scale bar indicates nucleotide substitutions per site. The marker indicates the CaKoV sequence detected in this study.

In the fox pooled sample PVI1/2021/ITA, the SISPA/ONT strategy allowed the recovery of three contigs of 682, 1254, and 2823 nt in length that were assembled against the prototype strain (NC001918) of the species *Kobuvirus aichi*, genus *Kobuvirus*. The three contigs covered the partial VP0, VP1, 2B, 3D, and the complete VP3, 2C, 3A, 3B, and 3D coding regions. Upon sequence analysis, the obtained sequences showed 89.4–95.6% nt identities to the canine kobuvirus (CaKoV) sequences available in the database. In the phylogenetic tree, based on the nt sequence of the partial RdRp gene strain, CaKoV/fox/PVI1/2022/ITA was segregated within the species *Kobuvirus aichi*, falling into the Aichivirus A2 clades and grouped with CaKoVs previously identified in Italy in a roe deer, in a badger, and in a porcupine [59] (Figure 4).

## 4. Discussion

In the present study, we utilized a combination of consensus molecular diagnostics and third-generation sequencing technologies to explore the circulation of selected viral pathogens among wild carnivore populations in Northwestern Italy.

The presence of CPV-2 and FPV in free-ranging wild carnivores underscores the persistent threat that these viruses pose to wildlife health and conservation efforts. In our analyses, CPV-2 strains were identified in wolves, and an FPV strain was detected in a Eurasian badger, confirming that both viruses are present in ecosystems with multiple host species [4,68,69]. Previous studies have consistently indicated that wild canids and other carnivorous species are vulnerable to CPV-2/FPV infection [18,69], and our results support the idea that wild carnivores may not only act as spillover hosts but could also serve as supplementary reservoirs for these viruses [13,14,70]. The overall prevalence (2.9%) of CPV-2/FPV DNA detected in this survey was low, aligning with the sporadic occurrence of protoparvoviruses reported in other wildlife populations [5,71]. On sequence analysis of the VP2 gene, key amino acid markers in the viral capsids that are known to influence CPV host range and antigenicity were conserved, in particular, changes at the VP2 position 426 (Asn to Glu), defining the CPV-2c antigenic type, and at position 297 (Ser to Ala), characteristic of the CPV-new 2a lineage [46,72]. While the biological relevance of some capsid mutations remains unclear, sequencing and monitoring these changes in wild-host viruses is crucial [73,74]. In detail, strains CPV-2/Wolf/LI6/2021/ITA and CPV-2/Wolf/LI8/2021/ITA clustered with Italian CPV-new 2a strains identified in puppies illegally imported into the Friuli Venezia-Giulia region between 2018 and 2021 [22]. The strain CPV-2/Wolf/LI9/2022/ITA formed a cluster with the CPV-2c strains identified in dogs in Italy [22,51], in China (unpublished), in Nigeria [75], in Thailand in the brain of a dog [54], and in a wolf fecal sample in Italy [22]. The detection of a CPV-new 2a variant in wolves is particularly noteworthy, showcasing ongoing viral evolution that echoes patterns observed in domestic dog populations [76]. This result corroborates previous research indicating that novel CPV-2 variants can swiftly disseminate among canid populations, both domestic and wild, facilitated by shared habitats, overlapping prey ranges, and direct or indirect interactions among sympatric carnivores [22]. Furthermore, the high genetic similarity between our wolf CPV-2 isolates and those found in domestic dogs points to a persistent bidirectional flow of parvovirus strains between wild and domestic hosts [69,71].

Furthermore, the detection of an FPV strain in a badger underscores the extensive host range of the members of the *Protoparvovirus carnivoran1* species. While FPV is traditionally associated with felids, it has been identified in various non-felid carnivores, including canids and mustelids [13,14]. Our discovery of FPV in a badger aligns with earlier reports, indicating that Eurasian badgers may serve as susceptible hosts and potential reservoirs for FPV [13,22,56]. Molecular analysis of the FPV/Badger/TI6/2022/ITA strain demonstrated high nucleotide identity with FPV strains circulating among diverse hosts globally, including domestic cats [46,77], as well as wild carnivores [15,22,24,50,56,57]. This suggests that the FPV strain present in the badger may have been acquired through shared ecological spaces between felids and other susceptible species [50]. Notably, the FPV VP2 sequence from the badger exhibited amino acid substitutions (101-Ile to Thr and 103-Val to Ala). While the functional impact of these changes is uncertain, their occurrence hints at adaptive variation as FPV jumps into new hosts, underscoring the genetic plasticity of FPV in wildlife [15,78]. Although the implications of these mutations remain uncertain, their occurrence emphasizes the genetic plasticity of FPV in adapting to new hosts [69,70]. The role of wild mustelids, such as badgers, as hosts for FPV merits further investigation, especially considering that badgers inhabit areas overlapping with domestic cat and wild canid territories, thereby potentially exposing them to a variety of parvoviruses in the environment [14,18,79].

We detected CAdV-1 DNA in an intestinal sample from a red fox. The detection of CAdV-1 in a fox is consistent with numerous studies indicating that red foxes serve as important reservoir hosts for this virus in wild populations. For instance, molecular surveys conducted in Italy and the UK have frequently identified CAdV-1 in free-ranging red foxes, with notable prevalence or seropositivity rates [21,29,33,58]. Conversely, wolves and other larger wild carnivores are less frequently reported to harbor CAdV-1, although cases have been documented [30,32]. In our survey, the negativity of other wild carnivores mirrors previous research in which CAdV-1 DNA was exclusively detected in foxes and not in sympatric wolf populations in southern Italy [33]. Sequence analysis of the fox strain (CAdV-1/Fox/VI9/2021/ITA) revealed the highest nucleotide identity to CAdV-1 strains previously isolated from domestic dogs and various European wild carnivores [29,30,31,32]. Phylogenetic analysis confirmed that our CAdV-1 strain clusters closely with other CAdV-1 isolates from both domestic dogs and wild carnivores, indicating that the virus circulating within fox populations is part of the globally distributed CAdV-1 lineage maintained through interspecies transmission [33]. The ongoing circulation of CAdV-1 among wild carnivores such as foxes has important implications for management. Although infections are often subclinical, these hosts can serve as reservoirs, facilitating spillover into other susceptible species, including unvaccinated domestic dogs or vulnerable wild canids [31,58]. Lethal outbreaks of CAdV-1 have been documented in species such as wolves [31] and bears [32], raising concerns that endemic CAdV-1 in reservoir hosts could pose a significant conservation threat. The absence of CAdV-2 in our samples aligns with prior studies that have sporadically detected this virus in wild carnivores. Occasional detections of CAdV-2 have been reported in foxes and wolves [21,35], but its role in wildlife epidemiology appears negligible.

The SISPA strategy, coupled with ONT metaviromic sequencing, facilitated the detection of CaCV in a wolf sample that also tested positive for CPV-2c. CaCV belongs to the family *Circoviridae*, genus *Circovirus*, species *Circovirus canine* [62]. CaCV was described for the first time in 2011 in six dog sera [63]. Since then, CaCV has been reported in dogs worldwide [64,80,81] and in wild carnivores, including arctic and red foxes [82,83,84], wolves and badgers [17,85,86]. CaCV has a worldwide distribution [64,80,87], although its ecology within wild animal populations remains poorly understood. Surveillance studies across Europe have identified the presence of CaCV DNA in arctic and red foxes [82,83,84], wolves, and badgers [17,85,86], as well as in other species such as jackals [88] and minks [89]. In particular, our strain is closely related to CaCV strains detected in spleen samples collected from wolves in Italy and in Canada [21,85], in liver samples from arctic foxes in Norway [82], spleen samples from foxes in Italy and Canada [85,88], and from serum samples of foxes with meningoencephalitis in the United Kingdom [83]. Prior investigations in Italy have reported the presence of CaCV DNA in wolf populations, with prevalence rates of 26.4% [86], 47.2% [17], and 50% [33], exhibiting significant regional variability. Such differences may reflect underlying ecological or sampling factors, including host density and exposure opportunities, influencing the circulation of CaCV in different geographical regions [21]. Nonetheless, our findings confirm that CaCV is capable of circulating among wild wolves in Northwestern Italy, thereby expanding the existing knowledge of its geographic distribution. In particular, CaCV DNA was identified in a wolf duodenal sample from Piemonte, an alpine region characterized by sympatric carnivore communities with possible overlap between wild and domestic canids. In a recent study, the presence of CaCV was documented in red foxes from Valle d’Aosta, one of the two regions involved in our investigation, demonstrating circulation in both alpine regions with strains clustered within the proposed Group 5 [65,66,88]. Although the pathogenic role of CaCV in free-ranging carnivores remains incomplete and most detections in wildlife occur in clinically non-evident animals, the virus has been documented in co-infections and associated with enteric or vascular syndromes in dogs [65,87]. This suggests a possible contribution of opportunistic transmission of disease under stressful conditions or exposure to multiple pathogens. Environmental transmission routes (e.g., fecal contamination of habitats or depletion of infected carcasses) are plausible in alpine ecosystems and may facilitate spread among sympatric carnivores.

We also identified CaKoV RNA in a red fox pooled from the Valle d’Aosta sample using the SISPA/ONT protocol. CaKoV is a member of the species *Kobuvirus aichi* in the genus *Kobuvirus* within the family *Picornaviridae* [90], first detected in dogs in the United States of America (USA) [91,92] and subsequently identified in domestic dogs worldwide [93,94,95,96,97]. CaKoV has also been reported in wild carnivore species, including foxes in Italy [98] and in Germany [99], wolves in Italy [100], golden jackal, side-striped jackal (*Canis adustus*), and spotted hyena (*Crocuta crocuta*) in Tanzania [101].

On sequence analysis, the CaKoV strain showed 94–98% nucleotide identity with strains identified in both domestic and wild hosts. Specifically, our strain exhibited close similarity to CaKoVs found in Italian roe deer and foxes, Arctic foxes in China, and domestic dogs in Asia [59,98,102,103]. Notably, CaKoV strains had previously been identified in red foxes and wolves within this geographic area [98,100], and our current findings confirm the circulation of this virus within the local wildlife population. Additionally, the high similarity to CaKoV strains of canine origin suggests that spillover from domestic dogs may serve as a potential source of infection for wild foxes, possibly via environmental contamination or scavenging of infected carcasses. Likewise, the detection of CaKoV in remote wildlife and even in non-canids, such as a roe deer and porcupine in the Toscana region, raises the possibility that CaKoV is maintained in nature through wildlife that can act as mechanical, passive carriers that could facilitate the introduction and spread of CaKoV in the environment [59]. The pathogenic significance of CaKoV in wild carnivores remains uncertain, as infections are often subclinical. The foxes included in this study were found deceased during passive surveillance, with no indication that CaKoV was the causative agent [93,94,100]. However, CaKoV could contribute to enteric disease under specific conditions or during co-infection with other enteropathogens, as suggested in domestic animals [92,104]. From a conservation and One Health perspective, the presence of CaKoV in wildlife underscores the complex microbial sharing between domestic and wild species. Including emerging pathogens such as CaKoV in wildlife, disease surveillance can enhance our understanding of their circulation and evolutionary dynamics.

Over the course of several decades, control programs have aimed to eradicate high-impact canine viruses through widespread vaccination, focusing also on CPV and CAdV-1. However, these pathogens persist within dog populations due to vaccination failures, the movement of infected animals from endemic regions, and their ongoing circulation in wildlife reservoirs [10,33,105]. Noteworthy, unvaccinated or poorly supervised dogs can serve as maintenance hosts and shed carnivore protoparvoviruses [105]. In Italian Alpine areas, field observations suggest that even pet dogs may contribute to viral persistence if they are not adequately vaccinated or allowed to roam freely [106]. Furthermore, phylogenetic and geographic data show frequent interactions between domestic and wild carnivores. FPV and CPV-2 strains have been found in wildlife, and regions with CPV-2-positive wild canids are more likely to see cases in owned dogs [13,22,105]. Similarly, European studies reveal that red foxes could carry CAdV-1 and may infect dogs, underscoring the bidirectional risk at the domestic–wildlife interface [29,107]. Collectively, these findings emphasise the need for targeted management strategies, including maintaining high vaccination coverage, restricting free-ranging behavior of local dogs, and managing feral cat colonies through population and vaccination efforts to curb transmission between wildlife and domestic animals.

## 5. Conclusions

Our findings provide molecular and phylogenetic evidence of the circulation of diverse viral pathogens, including CPV-2, FPV, CaCV, CAdV-1, and CaKoV, among wild carnivores in Northwestern Italy. Although the overall prevalence was low, these results emphasize the persistent risk of cross-species transmission and pathogen spillover between domestic animals and wildlife. Continued molecular surveillance and collaboration among veterinary, conservation, and public health sectors will improve our ability to detect and mitigate viral threats at the wildlife–domestic animal interface, thereby contributing to the preservation of biodiversity and animal health.

## Figures and Tables

**Table 1:** Viral pathogens detected in wild carnivore species assessed in this study with molecular investigations.

Animal Species Tested	No. of Animals	Virus % (Positive/Total)		Total % (Positive/Total)
		**FPV/CPV-2**	**CAdV-1/CAdV-2**	
Wolf (*Canis lupus*)	47	6.4% (3/47)	0% (0/47)	6.4% (3/47)
Fox (*Vulpes vulpes*)	55	0% (0/55)	1.8% (1/55)	1.8% (1/55)
Stone marten (*Martes foina*)	15	0% (0/15)	0% (0/15)	0.0% (0/15)
Eurasian badger (*Meles meles*)	23	4.3% (1/23)	0% (0/23)	4.3% (1/23)
**Total %** **(Positive/Total)**	**140**	**2.9% (4/140)**	**0.7% (1/140)**	**3.6% (5/140)**

**Table 2:** Statistics of ONT sequencing data.

Strain ID	Virus Species	Virus Name	Reads Assembled	Total Reads	No. of Contigs	Coverage Length (nt)	Reference Sequence
CPV-2/Wolf/LI6/2021/ITA	*Protoparvovirus carnivoran1*	CPV-2a	131,239	188,291	1	4492	NC001539
CPV-2/Wolf/LI8/2021/ITA		CPV-2a	489,633	886,740	1	4492	NC001539
CPV-2/Wolf/LI9/2022/ITA		CPV-2c	831,287	1,191,652	1	4492	NC001539
FPV/Badger/TI6/2022/ITA		FPV	344,382	399,962	1	4591	NC001539
CaCV/Wolf/LI9/2022/ITA	*Circovirus* *canine*	CaCV	588	1,191,652	1	2063	NC020904
CaKoV/Fox/PVI1/2021/ITA	*Kobuvirus aichi*	CaKoV	2630	92,178	3	4759	NC001918
CAdV-1/Fox/VI9/2021/ITA	*Mastadenovirus canidae*	CAdV-1	7356	57,335	8	17,320	NC001734

## Data Availability

Data supporting the results of this study are available from the corresponding author upon reasonable request. Sequence data have been deposited in GenBank and are openly accessible.

## Supplementary Materials

The following supporting information can be downloaded at: https://www.mdpi.com/article/10.3390/vetsci12090814/s1, Figure S1: (**a**) Polymerase chain reaction (PCR) amplification for members of the species *Protoparvovirus carnivoran1* [37]. Line M: BenchTop 1 kb DNA ladder; line 3, 4, 5, and 7: wolf positive samples corresponding to LI6, LI8, LI9, and TI6, respectively. (**b**) PCR amplification for CAdV-1/CAdV2 [38]. Line M: BenchTop 1 kb DNA ladder; line 4: fox positive sample (VI9).
