# Supplementary material for: Molecular Survey for Major Canine Enteric Viral Pathogens in Wild Carnivores, Northwestern Italy
Source: Vet Sci. 2025 Aug 26;12(9):814. doi: 10.3390/vetsci12090814 (PMC12474093; doi:10.3390/vetsci12090814)
Supplement: Supplementary file 1 [file vetsci-12-00814-s001.zip › vetsci-3812446-supplementary.pdf]

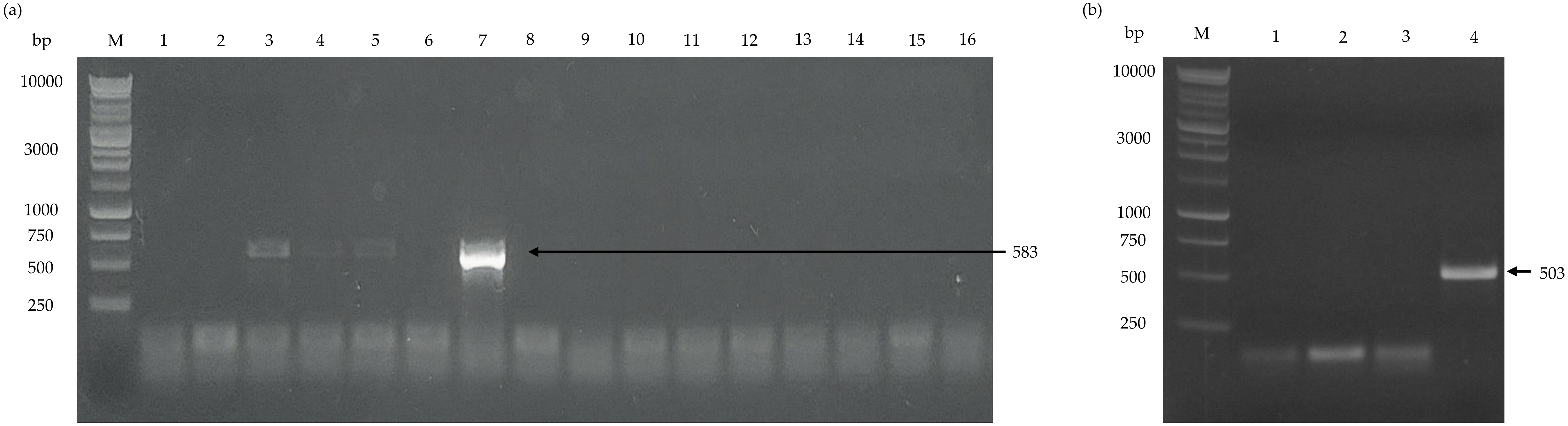

Figure S1. (a) Polymerase chain reaction (PCR) amplification for members of the species *Protoparvovirus carnivoran1* [37]. Line M: BenchTop 1 kb DNA ladder; line 3, 4, 5, and 7: wolf positive samples corresponding to LI6, LI8, LI9 and TI6, respectively. (b) PCR amplification for CAdV-1/CAdV2 [38]. Line M: BenchTop 1 kb DNA ladder; line 4: fox positive sample (VI9).
